# Unknown Risk on the Farm: Does Agricultural Use of Ionophores Contribute to the Burden of Antimicrobial Resistance?

**DOI:** 10.1128/mSphere.00433-19

**Published:** 2019-09-25

**Authors:** Alex Wong

**Affiliations:** a Department of Biology, Carleton University, Ottawa, Ontario, Canada; U.S. Centers for Disease Control and Prevention

**Keywords:** ionophore, cross-resistance, coselection, veterinary microbiology, agriculture, monensin

## Abstract

Ionophores are the second most widely used class of antibiotic in agriculture, with over 4 million kilograms sold in the United States in 2016. Because ionophores are not used in humans, it is widely assumed that their agricultural use will not impact human health. Consequently, these drugs have not been subject to the same regulations as medically important antibiotics. Here, I argue that the current evidence base is insufficient to conclude that ionophores do not contribute to human relevant antimicrobial resistance. It is unclear whether ionophore resistance can result in cross-resistance to medically important antibiotics. Moreover, recent evidence suggests that ionophore use may coselect for resistance to vancomycin in some cases. Systematic investigation of the consequences of agricultural ionophore use for human health is therefore imperative.

## Abstract (precis)

Ionophores are the second most widely used class of antibiotic in agriculture, with over 4 million kilograms sold in the United States in 2016. Because ionophores are not used in humans, it is widely assumed that their agricultural use will not impact human health. Consequently, these drugs have not been subject to the same regulations as medically important antibiotics.

## PERSPECTIVE

Antimicrobial resistance (AMR) is a serious and growing challenge in the treatment of infectious disease, with single-drug- or multidrug-resistant varieties of most major pathogens circulating at appreciable frequencies. It is increasingly acknowledged that AMR management efforts need to look beyond the clinic, since antimicrobials, AMR pathogens, and resistance genes may be found throughout the natural and built environments (1–4). For example, while human medicine is the most obvious source of clinically relevant resistance, the widespread and massive use of antibiotics in animal agriculture likely plays an important role in the persistence and spread of AMR. Consequently, national and international AMR strategies embrace the One Health paradigm (5–8), which highlights the need to consider agricultural and environmental contributors to AMR.

Recently, the use of antibiotics in farm animals has come under increased scrutiny (7, 9) since resistance that arises in animals may ultimately impact human health (10). In the Netherlands, for example, a national program to limit the veterinary use of antibiotics has seen sales drop by over 60% between 2009 and 2017 (11), largely with concomitant reductions in the prevalence of AMR among bacterial isolates from farm animals (11, 12). In Canada, a growth promotion ban on medically important antibiotics (MIAs) has been in place since 1 December 2018 (9). Regulation of veterinary antibiotics has primarily focused on MIAs, i.e., those that are used, or that belong to drug classes that are used, in treating human infectious disease (see, e.g., reference 13 and https://www.canada.ca/en/health-canada/services/drugs-health-products/veterinary-drugs/antimicrobial-resistance/categorization-antimicrobial-drugs-based-importance-human-medicine.html).

Other drugs, such as ionophores, are used solely in animals and are thus not considered medically important by major regulatory agencies (7, 14) (https://www.canada.ca/en/health-canada/services/drugs-health-products/veterinary-drugs/antimicrobial-resistance/categorization-antimicrobial-drugs-based-importance-human-medicine.html). They have thus escaped some growth promotion bans (https://www.canada.ca/en/public-health/services/antibiotic-antimicrobial-resistance/animals/actions/responsible-use-antimicrobials.html). Nonetheless, ionophores are the second most widely used class of antibiotic in animals in both the United States and Canada (14, 15), with 4.6 million kg of ionophores sold in the United States in 2016 (14); their use is likely to increase as medically important drugs are withdrawn from animal use. In light of their heavy use in agriculture, it is imperative that we understand the contribution of ionophores to the overall burden of AMR. Here, I argue that the current evidence base is insufficient to conclude that ionophores do not contribute to human relevant AMR.

**FIG 1:**
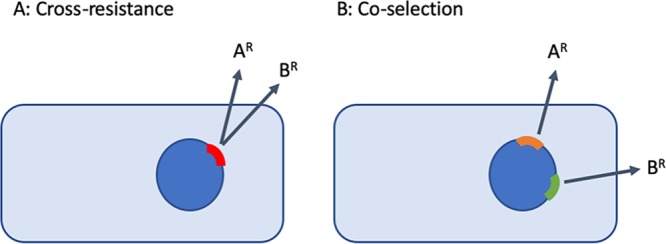
(A) A single mutation or gene may confer cross-resistance to two or more drugs (here, drugs “A” and “B” as hypothetical examples). (B) Alternatively, if separate resistance (R) mutations or genes are linked, selection with one drug will coselect for resistance to a second drug.

While they are not used in humans due to toxicity, the use of ionophores may still carry risk, owing to the possibility of cross-resistance or coselection (Fig. 1). Resistance to any drug may be accompanied by cross-resistance to other antibiotics. For example, animal use of avoparcin was halted in Europe in 1997 due to concerns of vancomycin cross-resistance in human zoonotic pathogens (16). Vancomycin and avoparcin are structurally similar glycopeptides, so cross-resistance between these drugs is perhaps unsurprising. However, cross-resistance can even arise toward chemically unrelated drugs. The multiple antibiotic resistance (Mar) phenotype in Escherichia coli, for instance, results from regulatory mutations that increase efflux and decrease cell permeability, resulting in resistance to several distinct drug classes (quinolones, phenicols, and some β-lactams [17]). Similarly, vancomycin-resistant enterococci (VRE) selected in the presence of the antiseptic chlorhexidine sometimes show cross-resistance to the antibiotic daptomycin, possibly due to alterations to membrane phospholipids (18). Thus, the use of animal-only drugs could in principle lead to cross-resistance to MIAs through shared resistance mechanisms. Notably, both the U.S. Food and Drug Administration (FDA) and Health Canada (HC) explicitly consider cross-resistance in categorizing the safety risks of drugs for veterinary use (13) (https://www.canada.ca/en/health-canada/services/drugs-health-products/veterinary-drugs/antimicrobial-resistance/categorization-antimicrobial-drugs-based-importance-human-medicine.html).

Coselection can occur if resistance genes for several drugs are genetically linked. The use of any one of the drugs to which an organism is resistant will increase the frequency of all linked AMR genes, even those that are not under direct selection. For example, experimental supplementation of animal feed with sulfonamides (a drug class of only “medium importance” to human health, according to Canadian regulations) can coselect for carbapenem resistance in Klebsiella pneumoniae (19). Genetic linkage can occur because of physical linkage, for example, when two resistance genes are on the same plasmid (20, 21). Importantly, however, physical linkage is not necessary for coselection: so long as rates of horizontal gene transfer are not too high, all genes in the genome of an asexual organism are genetically linked. Coselection is thought to underlie the failure of drug restriction protocols to reduce the prevalence of AMR in several cases (22–24). Again, the use of animal-only drugs could contribute to resistance to MIAs if resistance genes to these drugs are linked.

## IONOPHORE RESISTANCE, CROSS-RESISTANCE, AND COSELECTION

Ionophores are a diverse class of antibiotics, largely produced by bacteria in the genus Streptomyces (25, 26). Structurally, ionophores used in agriculture are carboxylic polyethers, a subclass of polyketides. Ionophores bind cations and transport them across the cell membrane, disrupting cellular ion balance (reviewed in reference 27). Monensin, for example, is an antiporter that transports K^+^ out of, and Na^+^ and H^+^ into, the cell. These changes are ultimately thought to disrupt proton motive force (28). Other ionophores have different ion preferences; lasalocid, for example, prefers divalent metal cations and organic bases (26). Ionophores are primarily active against Gram-positive organisms, although there are a few exceptions (see, e.g., reference 29).

Several ionophores, including lasalocid, monensin, narasin, and salinomycin, are routinely administered to cattle and/or pigs for growth promotion. Furthermore, these same drugs, as well as maduramicin, are used for the prevention of coccidiosis in poultry and other farm animals (30–33). Data are not readily available on the distribution of ionophore use by animal species. For example, the FDA only reports on ionophore use aggregated across all animal species, owing to confidentiality constraints, whereas the use of medically important antibiotics is broken down according to animal species (cattle, swine, chicken, turkey, and other [14]). Growth promotion in cattle is thought to occur through alterations to ruminal fermentation (25, 34), presumably due to changes in the gut microbiome. Ionophores may also have direct impacts on the host’s metabolism and physiology (35).

For the most part, mechanisms of ionophore resistance are not well understood (Table 1). The one known mechanism comes from Streptomyces longisporoflavus, the bacterium that produces the ionophore tetronasin. By expressing an S. longisporoflavus genomic library in Streptomyces lividans (which is partially tetronasin sensitive), Linton et al. identified a putative ABC transporter that confers tetronasin resistance (36). Thus, it is thought that ABC transporter-mediated efflux is at least partially responsible for intrinsic tetronasin resistance in *S. longisporoflavus.* Related ABC transporter genes are associated with narasin resistance in VRE isolated from Swedish broiler chickens (37, 38), although knockout/knock-in studies have not confirmed this finding.

**TABLE 1:** Known and putative mechanisms of ionophore resistance

Antibiotic	Mechanism	Organism	Genetic basis known?	Reference
Tetronasin	Efflux	*Streptomyces longisporoflavus*	Yes	36
Narasin	Efflux (putative)	VRE	Yes	38
Tetronasin	Permeability	*Prevotella ruminicola*	No	29
Monensin	Cell wall thickening	*Enterococcus spp.*, *C. perfringens*	No	39
Monensin	Increased extracellular polysaccharide	*C. aminophilum*	No	40

Ionophore resistance can be selected in the lab by serial passaging in the presence of antibiotic (29, 39). In Prevotella ruminicola (previously Bacteroides ruminicola), a rare Gram-negative bacterium that is tetronasin sensitive, phenotypic analyses suggested that evolved tetronasin resistance was caused by changes in cell envelope permeability (29). Similarly, monensin-resistant isolates of Enterococcus spp. and Clostridium perfringens had thicker cell walls than did unexposed controls (39), and monensin adaptation in Clostridium aminophilum was accompanied by changes in several cell wall/envelope characteristics (40). Thus, there is phenotypic evidence that acquired ionophore resistance can be mediated by changes to the cell wall and/or envelope. To date, however, mutations associated with acquired ionophore resistance have not been identified. I note that Simjee et al. argued that monensin resistance was not mutation based, since resistance decreased after several weeks of culture in the absence of drug (39). This is not a compelling argument, for if monensin resistance is costly, compensatory mutations or back mutations arising during drug-free culture could reduce levels of resistance.

The prevalence of ionophore resistance is difficult to assess, since there are no clinical breakpoints for resistance. Moreover, large-scale surveys have not been carried out given the animal-only use of ionophores. A few attempts have been made, however. Aarestrup et al., defining resistance in terms of a markedly higher MIC, found up to 6% monensin resistance in Staphylococcus hyicus and *Enterococcus* spp. in pigs (41; see also reference 42). Other instances of circulating ionophore resistance have been described but do not provide estimates of prevalence; for example, decreased narasin sensitivity was found for some poultry-derived VRE strains in Sweden (37).

Houlihan and Russell (43) are frequently cited in support of the claim that ionophores do not cause meaningful cross-resistance to MIAs (44, 45). Here, cross-resistance toward 16 different drugs, mostly MIAs, was measured in monensin- or lasalocid-resistant cultures of Clostridium aminophilum. Cross-resistance was only noted for bacitracin, and not for any other drug, suggesting that cross-resistance is not an issue. I suggest that these results should be interpreted with caution, as sample sizes in this study were small (*n* = 3), cultures were selected in ionophore for only a single growth cycle, and drug susceptibility testing appears to have been carried out on mixed cultures rather than on isolated genotypes. Thus, it is inappropriate to conclude from this work that ionophore resistance does not present risks to human health.

Few other studies have examined cross-resistance between ionophores and MIAs. Tetronasin resistance in *P. ruminicola* was accompanied by a modest increase in resistance to avoparcin, a glycopeptide with similarities to vancomycin (29); no other medically relevant drugs were tested in this study. Notably absent from the literature are studies on cross-resistance in human pathogens. The observation that ionophore resistance is sometimes accompanied by thickening of the cell wall (Table 1) does raise the possibility of cross-resistance, since cell wall thickening is thought to contribute to resistance to some MIAs (46, 47).

The prospect of ionophore-mediated coselection for AMR has been investigated in several animal systems. Multiple studies in poultry have found that ionophore feed supplementation has mixed effects on AMR, with decreases in the prevalence of some resistances, increases in the prevalence of others, and no effect on others (48–51). It should be noted, however, that most of these studies measured AMR in specific Gram negatives (e.g., E. coli). Given the specificity of ionophores toward Gram positives, I suggest that Gram-positive indicator organisms, or metagenomic analyses, will be more informative regarding the clinical relevance of ionophore use. Along these lines, a recent metagenomic study found that monensin treatment of cattle had little to no effect on the population of antibiotic resistance genes (ARGs) in the gut (52).

There is, however, evidence of an emerging association between narasin resistance and vancomycin resistance in Swedish broiler chickens, with a putative narasin resistance ABC transporter located on the same plasmid as a *vanA* gene cluster, which confers vancomycin resistance in VRE (38). This finding raises the possibility that vancomycin resistance could be maintained in animal populations not because of treatment with vancomycin or related compounds but because of ionophore use. In a recent search of the nucleotide database at the NCBI, I found 15 strains of *Enterococcus* spp. bearing identical or near-identical matches to both genes encoding this putative narasin transporter. These strains came from around the world, including the United States, Scandinavia, and Taiwan, indicating a global distribution. Notably, these strains were derived from both animal and human sources. Linkage to *vanA* was present in some, but not all, strains. Coselection is therefore a real possibility, and more work will be required to assess its actual impact on AMR in human pathogens.

## CONCLUSIONS

Widespread veterinary use of ionophores has been assumed to be safe for humans, largely on the grounds that this class of drugs is not used in human medicine. However, the risks posed by cross-resistance to MIAs, and by coselection between ionophores and MIAs, have not been adequately determined. The UK Review on Antimicrobial Resistance, which culminated in the seminal O’Neill report, recognized this dearth of evidence in writing, “It is clear to us, however, that more research is needed to ensure that ionophores, and other widely used antimicrobials, are not contributing to resistance problems” (53). There is thus a clear and urgent need to systematically investigate the contribution of ionophores to the burden of antimicrobial resistance.
